# *Verbena officinalis* Verbenaceae (Lamiales): a new plant model system for phyllotaxis research

**DOI:** 10.1007/s10265-021-01288-2

**Published:** 2021-04-08

**Authors:** Beata Zagórska-Marek, Magdalena Turzańska, Klaudia Chmiel

**Affiliations:** Institute of Experimental Biology, University of Wroclaw, Kanonia Str. 6/8, 50-328 Wrocław, Poland

**Keywords:** Computer simulations, Morphogenesis, Phyllotactic diversity, Plant development, Shoot apical meristem, Vervain

## Abstract

Phyllotactic diversity and developmental transitions between phyllotactic patterns are not fully understood. The plants studied so far, such as *Magnolia, Torreya* or *Abies*, are not suitable for experimental work, and the most popular model plant, *Arabidopsis thaliana,* does not show sufficient phyllotactic variability. It has been found that in common verbena (*Verbena officinalis* L.), a perennial, cosmopolitan plant, phyllotaxis differs not only between growth phases in primary transitions but also along the indeterminate inflorescence axis in a series of multiple secondary transitions. The latter are no longer associated with the change in lateral organ identity, and the sequence of phyllotactic patterns is puzzling from a theoretical point of view. Data from the experiments in silico, confronted with empirical observations, suggest that secondary transitions might be triggered by the cumulative effect of fluctuations in the continuously decreasing bract primordia size. The most important finding is that the changes in the primary vascular system, associated with phyllotactic transitions, precede those taking place at the apical meristem. This raises the question of the role of the vascular system in determining primordia initiation sites, and possibly challenges the autonomy of the apex. The results of this study highlight the complex relationships between various systems that have to coordinate their growth and differentiation in the developing plant shoot. Common verbena emerges from this research as a plant that may become a new model suitable for further studies on the causes of phyllotactic transitions.

**Supplementary Information:**

The online version contains supplementary material available at 10.1007/s10265-021-01288-2.

## Introduction

The regular distribution of lateral organs in plant axial systems, called phyllotaxis, has intrigued scientists for a long time. Interesting examples of this universal phenomenon have been found among brown algae (Peaucelle and Couder 2016), leafy bryophytes (Zagórska-Marek et al. 2018), clubmosses (Gola 1996; Yin and Meicenheimer 2016), and seed plants (Yin et al. 2011; Zagórska-Marek 1985; for review see Gola and Banasiak 2016). The best known and most common are whorled or spiral phyllotactic patterns, and among the latter, the main Fibonacci pattern prevails. However, over the years, quite a large body of empirical evidence has accumulated, indicating that phyllotactic diversity, especially among spiral patterns, is more complex than originally thought, and all patterns may transform developmentally, one into another (Zagórska-Marek 1985, 1994).

Transformations of phyllotaxis are often associated with a new phase of development, during which the identities of organs change. Opposite cotyledons may be followed by spirally arranged foliage leaves. While vegetative phyllotaxis is often spiral, floral phyllotaxis is mostly whorled. The converse situation is exemplified by all Calycanthaceae (Staedler et al. 2007). The bijugate phyllotaxis of the *Torreya* shoot becomes decussate within the domain of cataphylls formed at the end of each growing season (Banasiak and Zagórska-Marek 2006). The *Magnolia* flower has its perianth elements in trimerous whorls, but the stamens and carpels are spirally arranged. However, phyllotactic transitions may occur even when organ identity does not change, as in the vegetative shoots of *Abies balsamea* and in the gynoecia of *Magnolia* (Zagórska-Marek 1985, 1994). Moreover, the phyllotaxis of the newly initiated leaf-like organs in reverted flowers of *Impatiens balsamina* does not return to the spiral mode, which is typical for this plant’s vegetative growth phase, but remains floral, i.e., whorled (Battey and Lyndon 1990). Petalody in *Magnolia stellata* shows that additional petals are not laid out in whorls but in spirals (Wróblewska et al. 2016). The last two cases indicate that the genetic mechanism specifying primordia distribution acts independently from that responsible for their identity.

What is then required for a change in phyllotaxis if not a new identity of the organ primordium? An intuitive guess is that it should be an appropriate change in its size relative to that of the shoot apical meristem (SAM). In the model plant *Arabidopsis thaliana,* the vegetative phyllotaxis of the leaves assembled into a rosette is spiral shaped, mirroring that of the cauline leaves and cryptic bracts on the inflorescence axis in the next phase of growth (Kang et al. 2003; Mueller-Xing et al. 2014). The changes in primordia identity and, most likely, their relative size between the two stages of growth is evidently insufficient for a change in phyllotactic pattern quality.

The results of computer modeling confirm that phyllotactic transitions occur when primordia size changes in a specific manner with respect to the size of the SAM’s organogenic surface (Douady and Couder 1996; Szpak and Zagórska-Marek 2011; Zagórska-Marek and Szpak 2008, 2016). In virtual magnolia, the changes in primordia size between the domains of the whorled trimerous perianth and spiral androecium explain the presence of trijugy, which is frequently encountered in real magnolia flowers (Zagórska-Marek 1994) but is rare among helical patterns in other plants. The computer simulations have also shown that when the change is slow, rather than abrupt as anticipated for the cases in which primordia identity changes, phyllotaxis remains qualitatively constant until the moment when small irregularities in primordia position, accumulating over a long period of time, introduce a new packing system (Zagórska-Marek and Szpak 2016). This suggests the importance of the initial primordia number in the history of the particular meristem and explains why phyllotaxis may sometimes change without a change in organ identity.

Other factors affecting phyllotaxis could be the putative signals sent acropetally to the apex from the differentiating primary vascular system. Composed entirely of leaf traces, the system is structurally related to phyllotactic order. Although there is some evidence supporting this hypothesis (Banasiak 2010; Larson 1975, 1977), it still awaits to be either confirmed or falsified unequivocally.

Finding a plant system with indeterminate growth and a relatively large number of lateral organs with the same identity that is suitable for molecular-genetic studies is not easy. This paper introduces common verbena (*Verbena officinalis* L.)*,* which not only meets these requirements but also exhibits multiple developmental changes in phyllotaxis that occur in a seemingly non-random manner. The course of these natural phyllotactic transitions will be confronted with the results of computer simulations. In addition to having all virtues of *A. thaliana*, the phyllotaxis of which is rather uniform (Dengler 2006; Kang et al. 2003), common verbena has one additional advantage that makes it especially attractive: it readily propagates vegetatively. Accepting *Verbena officinalis* as a new model plant may bring us closer to understanding the molecular genetics that underlies phyllotactic ontogenetic transformations.

## Material and methods

*Verbena officinalis,* common vervain, or common verbena, native to Europe according to the U.S. National Plant Germplasm System (2020) (https://npgsweb.ars-grin.gov/gringlobal/taxonomydetail.aspx?id=41164), is a cosmopolitan species widely distributed on nearly every continent. First, plants were collected for investigation from a few isolated stands in Lower Silesia, Poland near the villages of Luboradz and Gaworzyce. From their seeds, a new population was established in an open field in the Przecławice village, located in Trzebnickie Hills north of Wrocław, Poland. The plants here grow at 200 m above sea level. Western circulation prevails in the area, bringing arctic air masses in the winter and tropical ones in the summer. The coldest month is January (− 1 to − 3 °C), and the warmest is July (17 to 19 °C). The average annual air temperature varies from 7 to 9 °C. The average annual precipitation (lowest in the winter and highest in July) is 550–600 mm (source: https://trzebnica.pl/4761/1087/fauna-flora-i-klimat-winnej-gory.html).

The plants from the Przecławice population were compared with geographically isolated representatives of the same species growing in the south of Europe, near Vodice, Croatia.

The distribution patterns of the foliage leaves on the vegetative stem and bracts on the flowering shoot axis were examined, both along the circumference and vertically, using the Nikon SMZ 745 T stereomicroscope with an Invenio 5SII (DeltaPix, Smorum, Denmark) digital camera and the DeltaPix InSight software. Selected stem fragments with unique or transforming phyllotaxis were sectioned live with a razor blade, and these handmade sections were immediately photographed under an Olympus BX50 epifluorescence microscope with a UV (360–370 nm) excitation filter, equipped with an Olympus DP71 digital camera and the CellˆB software.

The tips of vegetative shoots and dense terminal spikes of flowering shoots were harvested, immediately placed in FAA fixative (formaldehyde[37%], ethanol [50%], and glacial acetic acid [99.5%] in a 5:90:5 proportion), degassed using a vacuum-suction glass flask, and fixed for 24 h. After being rinsed in 50% ethanol, they were dehydrated using an alcohol series (ethyl alcohol–butanol). From 100% butanol, they were moved in a fume cupboard through a series of butanol–paraffin mixtures with an increasing content of paraffin, eventually reaching pure paraffin. The spikes embedded in the paraffin blocks were sectioned transversely using a rotary microtome (RM 2135 Leica, Germany) of 7–9 μm thickness. The serial sections mounted on the glass covered with Haupt adhesive were de-paraffined using xylene under a fume hood, moved to 100% ethanol, and then rehydrated to 70% ethanol. They were subsequently stained with a safranin solution (1 g of Safranin O [Sigma S8884] in 100 ml of 50% ethanol) for 12 h. After progressing through the ethanol series with alcohol concentrations reaching up to 90%, they were stained in 0.1% Fast Green solution (1 g of Fast Green FCF [Sigma F-7252] in 100 ml of 92% ethanol) for 5–7 min. After being rinsed in 96% ethanol, they were transferred to isopropyl alcohol (3 changes for 110 min) and then mounted in Euparal [Euparal for microscopy mounting agent (Roth 7356.2)].

The sections were subsequently photographed, and on these images, the sizes of the apical meristems and their lateral organ primordia were assessed in both developmental phases of growth. In the first step, the apex diameter was determined at the level of the youngest primordium insertion. Then, the leaf insertion angles were measured using the application available at the following address: https://www.ginifab.com/feeds/angle_measurement/online_protractor.pl-pl.php. Finally, the percentages of the apex circumference, consisting of the angle values, were calculated.

The computer simulations of phyllotactic transitions were performed using the latest version of the computer program “Phyllotaxis” (Zagórska-Marek and Szpak 2016).

## Results

### General morphology of the plant

**Fig. 1:**
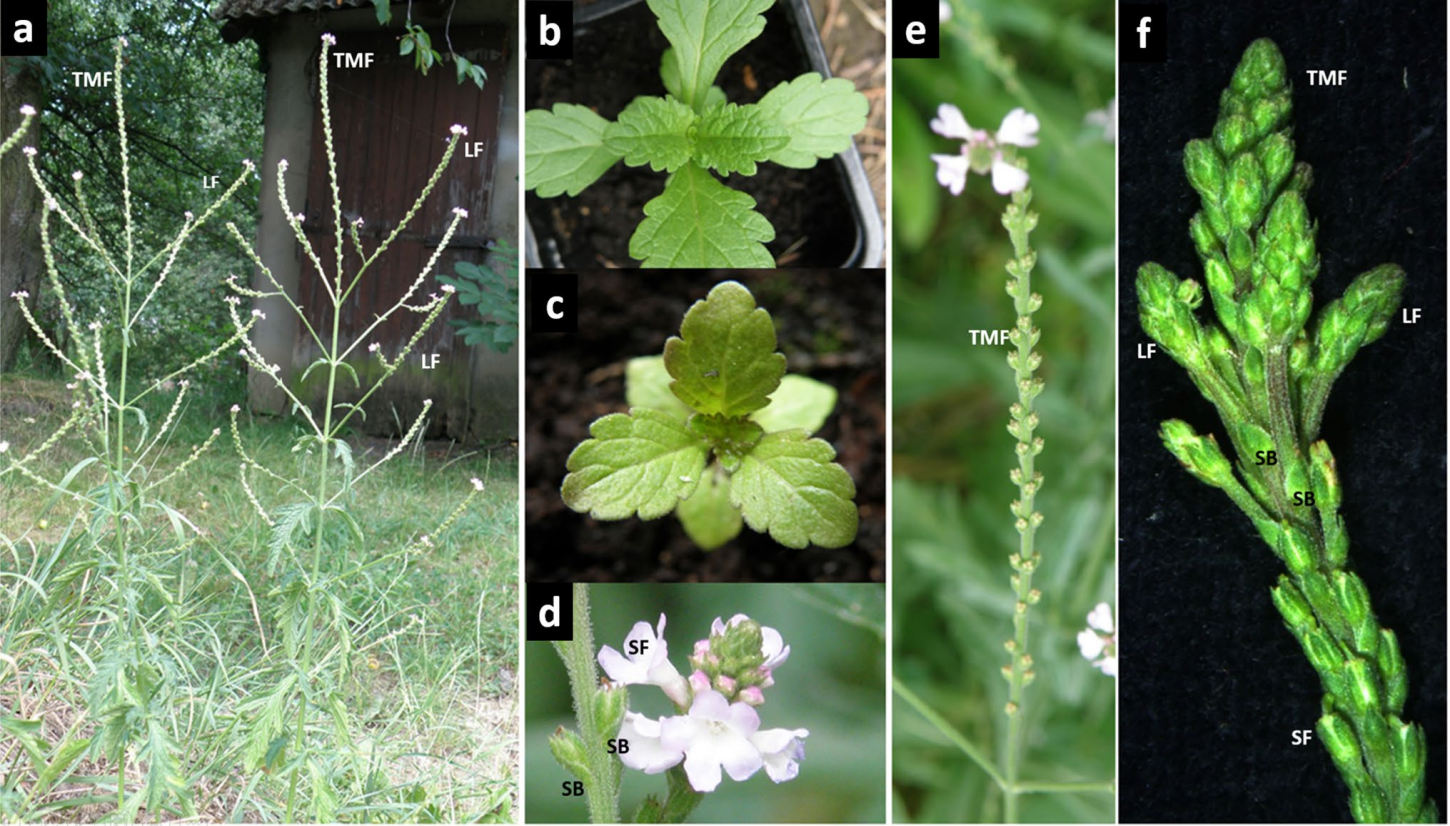
*Verbena officinalis*. **a**—two individual plants with characteristic opposite branching of the main shoots; the gradual decrease in the size of the foliage leaves along the main axis leads to emergence of scaly bracts (SB) in the terminal main florescence (TMF); single flowers (SF) develop in the axils of the bracts; the pattern repeats in each lateral co-florescence (LF); the number of nodes capable of forming leaves in the laterals decreases upward along the main stem—the last pair of laterals often grows out of the first pair of bracts; **b**—young plantlet with decussate phyllotaxis typical of vegetative growth phase; **c**—rare case of vegetative tricussate phyllotaxis; **d**—closeup of the terminal dense spike with flowers; **e**—elongated florescence axis; **f**—rare phenomenon of reversion resulting in the formation of multiple lateral florescences instead of single flowers in the axils of bracts

The shoot organization of fully developed, flowering plants appeared to be polytelic (Weberling 1983). Martinez et al. (1996) classified their branching form as heterothetic pleiobotryum. The erect main shoots, reaching 70 cm in height and terminating with main florescence, were highly branched (Fig. 1a). The lateral shoots (paracladia), which developed from the axils of the opposite leaves, iteratively recapitulated the architecture of the supporting shoot. Toward the tip, the vegetative status of these shoots, expressed by their ability to form foliage leaves, was gradually weakened. Like the main axis, they produced progressively reduced leaf blades, which ultimately transformed into scaly bracts at a certain level. The leaves differed from the bracts in the number of supplying vascular bundles—three for the leaf and one for the bract. From the axils of the first, often still opposite bracts, lateral florescences could emerge, but above them single flowers only formed in the bract axils. This gradual loss in vegetative potential resembled that observed in the architecture of *A. thaliana* wild-type (WT) plants*.* All flowering shoots exhibited indeterminate growth, regardless of whether they were terminal (main florescence) or lateral (co-florescence). They attained considerable length during one growing season, producing over 150 bracts. Their axils formed single flowers. Each of them, if pollinated, gave rise to a dry schizocarpic fruit, which split at maturity into four one-seeded mericarps. This made the number of seeds per florescence quite substantial. Elongation of the internodes between the consecutive bracts was delayed until the corolla of the pollinated flower wilted and fell off. As a result, each elongating and maturing axis ended with a small, dense spike of bracts, axillary flowers, and flower buds (Fig. 1d, e). One case of reversion was noted: instead of single flowers, the axillary meristems within the terminal spike produced multiple lateral florescences (Fig. 1f). The plants from the Przecławice population, observed for over a decade, were clearly perennial: fully developed shoots at the end of each growing season dried out, and after winter rest, new shoots grew from the underground rhizome.

### Developmental changes in phyllotaxis

Vervain phyllotaxis in the vegetative phase of growth was decussate (Fig. 1b). Sporadically, tricussate plants developed from the seedlings with three cotyledons (Fig. 1c). Vegetative phyllotaxis was stable, but with the onset of flowering, phyllotactic transitions occurred. The term ‘primary transitions’ was adopted for those between the vegetative and reproductive growth phase, and ‘secondary transitions’ was adopted for those in florescence. The achiral, whorled patterns of the foliage leaves, present also in paracladia, changed primarily into a chiral, helical arrangement of bracts on the florescence axis, and then secondary transitions took place, often in sequences of more than two consecutive patterns (multiple secondary transitions). Primary transitions in the decussate plants most frequently led to development of the main Fibonacci pattern. Sporadically, the main bijugy or Lucas pattern emerged (Tables 1, 2, 3). The primary transitions in tricussate plants resulted in Lucas phyllotaxis. The chirality of helical patterns expressed by the direction of the ontogenetic helix was, at this stage, established at random.

**Table 1:** General characteristics of phyllotactic patterns present in common verbena shoots

Phyllotactic pattern	Limiting value of divergence angle (in degrees)	Mathematical series (possible parastichy numbers)	Number of pattern elements on the shoot circumference
Decussate	180	2, 2	2
Main Fibonacci	137.5	1, 2, 3, 5, 8, 13	3
Tricussate	120	3, 3	3
Lucas	99.5	1, 3, 4, 7, 11	4
Tetracussate	90	4, 4	4
Second accessory	77.9	1, 4, 5, 9, 14	5
Main bijugy	137.5/2	2, 4, 6, 10	6

**Table 2:** Phyllotactic transitions recorded in common verbena shoots that involve a developmental change in the number of contact parastichies

Primary transitions	Secondary single transitions	Selected examples of secondary multiple transitions
2:2–2:3	2:3–3:3	2:3–3:5–3:4–4:4–4:5
2:2–2:4	2:3–3:4	2:3–3:3–3:4
2:2–3:4	3:3–2:3	2:3–3:4–4:5–4:6
3:3–3:4	3:3–3:4	2:3–3:4–2:4
	3:4–4:4	2:3–3:3–3:4–4:4–4:5
	3:4–2:4	2:3–2:4–4:4–4:5
	3:4–4:5	
	4:4–2:4	
	4:4–4:5	
	4:5–4:6

**Table 3:** Frequencies of phyllotactic patterns in 12 individual plants (I-XII) from the Przecławice population investigated 10 years ago and now; c/d—ratio of florescences with secondary multiple transitions to the total number of pattern cases

Genet	No. shoots	Primary transitions			c/d	Secondary multiple transitions					
		− 2:3	− 3:4	− 2:4		3:5	3:4	2:4	3:3	4:4	4:5
*Year 2010*											
I	152	126	1	0	25/65	12	24	15	7	4	3
	100%	83%	1%			18%	37%	23%	11%	6%	5%
II	96	84	1	0	11/29	7	12	3	5	1	1
	100%	87%	1%			24%	41%	10%	17%	3%	3%
III	35	22	0	0	13/39	9	13	6	4	1	6
	100%	63%				23%	33%	15%	10%	3%	15%
IV	114	87	5	1	21/68	14	26	16	6	2	4
	100%	76%	4%	1%		21%	38%	23%	9%	3%	6%
V	140	130	1	1	8/21	7	9	2	3	0	0
	100%	93%	1%	1%		33%	43%	9%	14%		
VI	70	58	7	1	4/11	3	4	3	1	0	0
	100%	83%	10%	1%		27%	36%	27%	9%		
*Year 2020*											
VII	88	68	3	1	16/33	14	6	10	2	1	0
	100%	77%	3%	1%		42%	18%	30%	6%	3%	
VIII	57	47	1	0	9/27	6	9	3	5	1	3
	100%	84%	2%			22%	33%	11%	18%	4%	11%
IX	48	37	1	0	7/31	9	10	3	4	3	2
	100%	79%	2%			29%	32%	10%	13%	10%	6%
X	28	21	2	1	4/14	5	3	3	1	1	1
	100%	75%	7%	4%		36%	21%	21%	7%	7%	7%
XI	97	82	2	0	13/35	9	13	4	9	0	0
	100%	84%	2%			26%	37%	11%	26%		
XII	50	45	0	0	5/15	7	4	0	4	0	0
	100%	90%				47%	27%		27%		

**Fig. 2:**
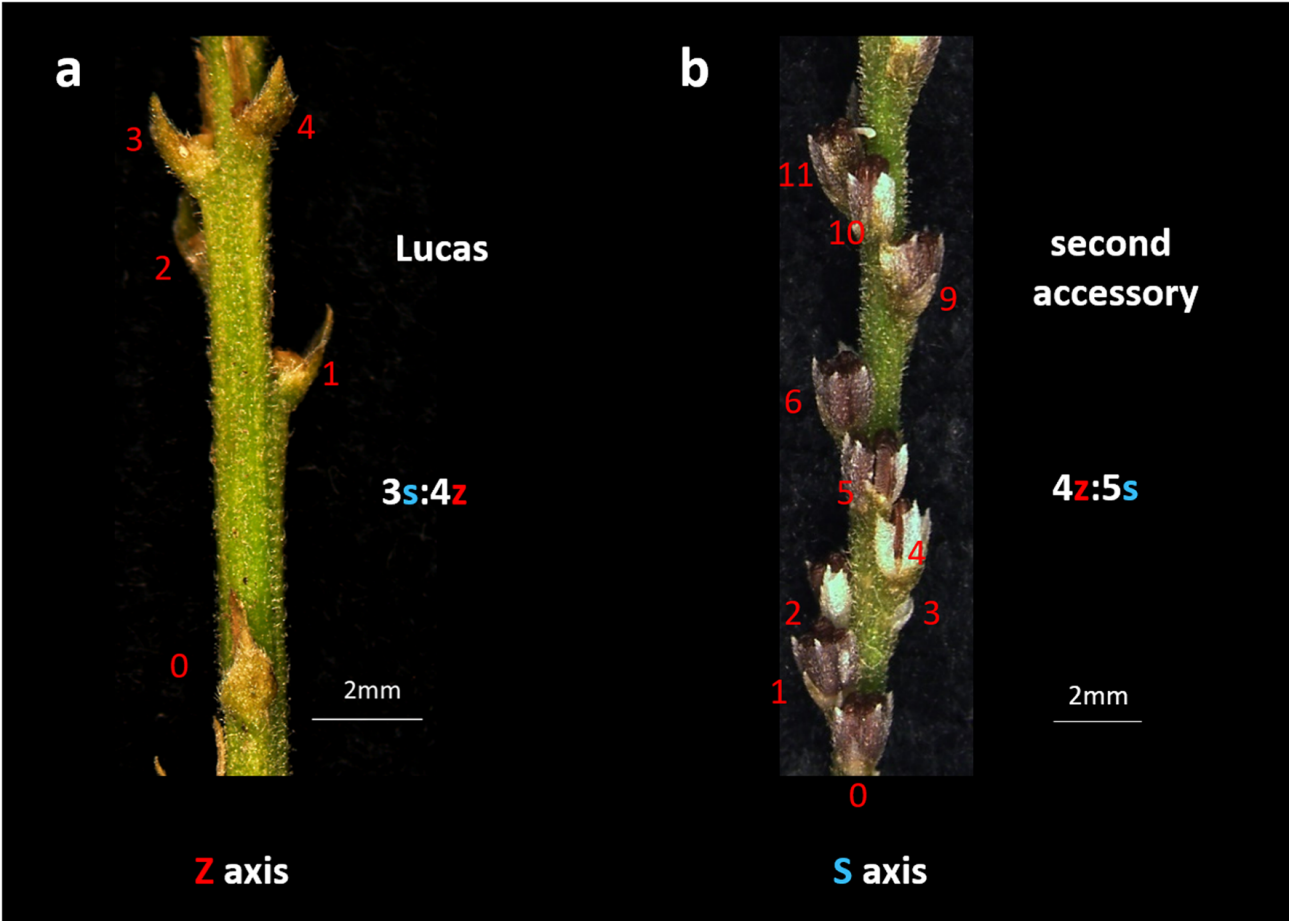
The side view of two elongated florescences with different phyllotaxis. There are four bracts on one revolution of the ontogenetic helix in the Lucas pattern (**a**) and five in the second accessory pattern (**b**). The developmental sequence of the consecutive bracts (numbered in red) shows the direction of the ontogenetic helix—counterclockwise (Z) in Lucas and clockwise (S) in the second accessory pattern

**Fig. 3:**
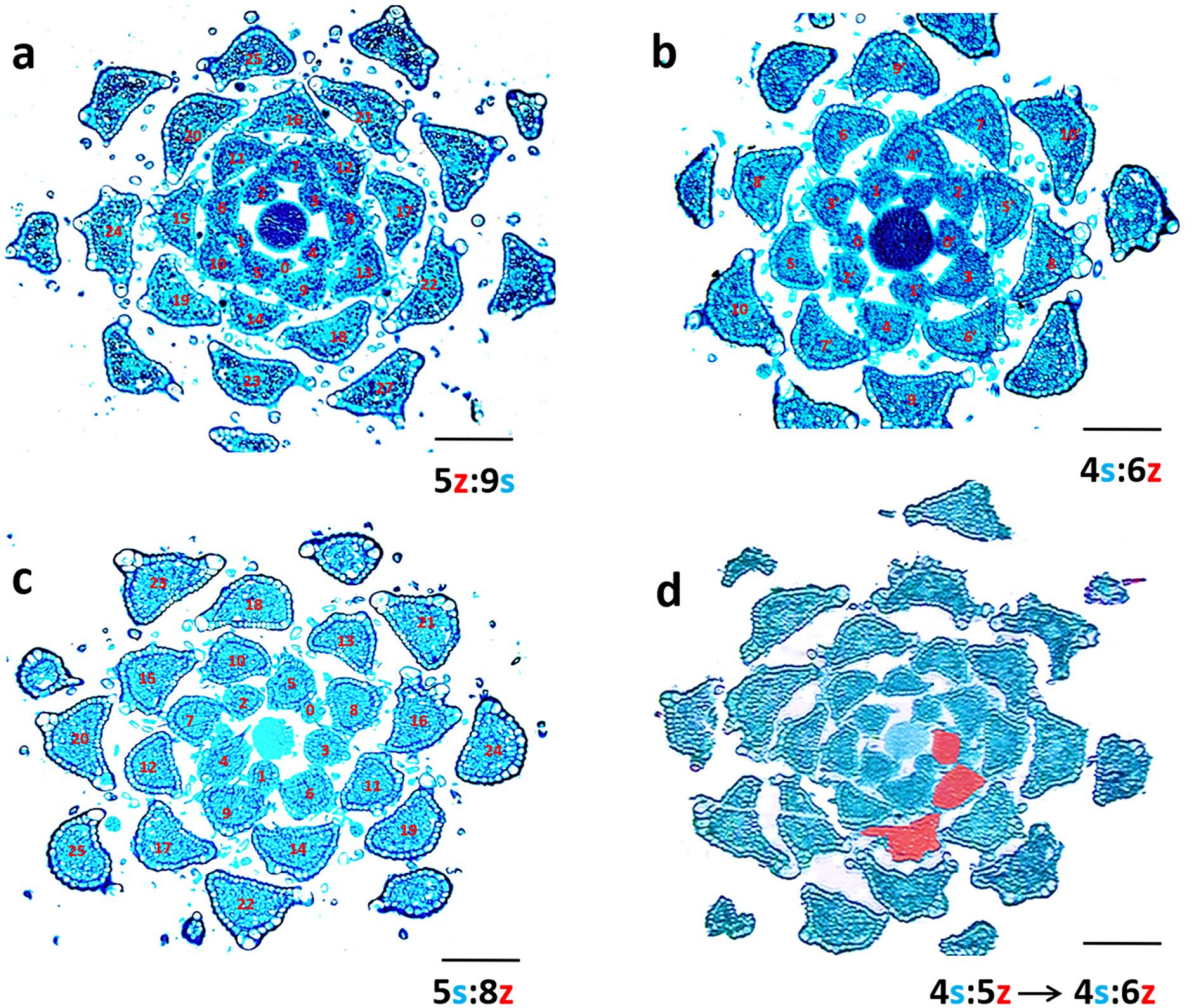
Phyllotactic “snowflakes” —terminal portions of four different florescences, sectioned transversely; high regularity of the bract setup allows identification of the phyllotactic pattern; the numbers at the bottom of each section pertain to imaginary spiral lines (parastichies) connecting bracts in a clockwise (blue S) or counterclockwise (red Z) direction; spiral phyllotaxis in each shoot is qualitatively different: **a**—second accessory, **b**—main bijugy, **c**—main Fibonacci, and **d**—second accessory transforming into main bijugy through the addition of one parastichy (colored red) in a set of five (**d**). Scale bars—120 µm

**Fig. 4:**
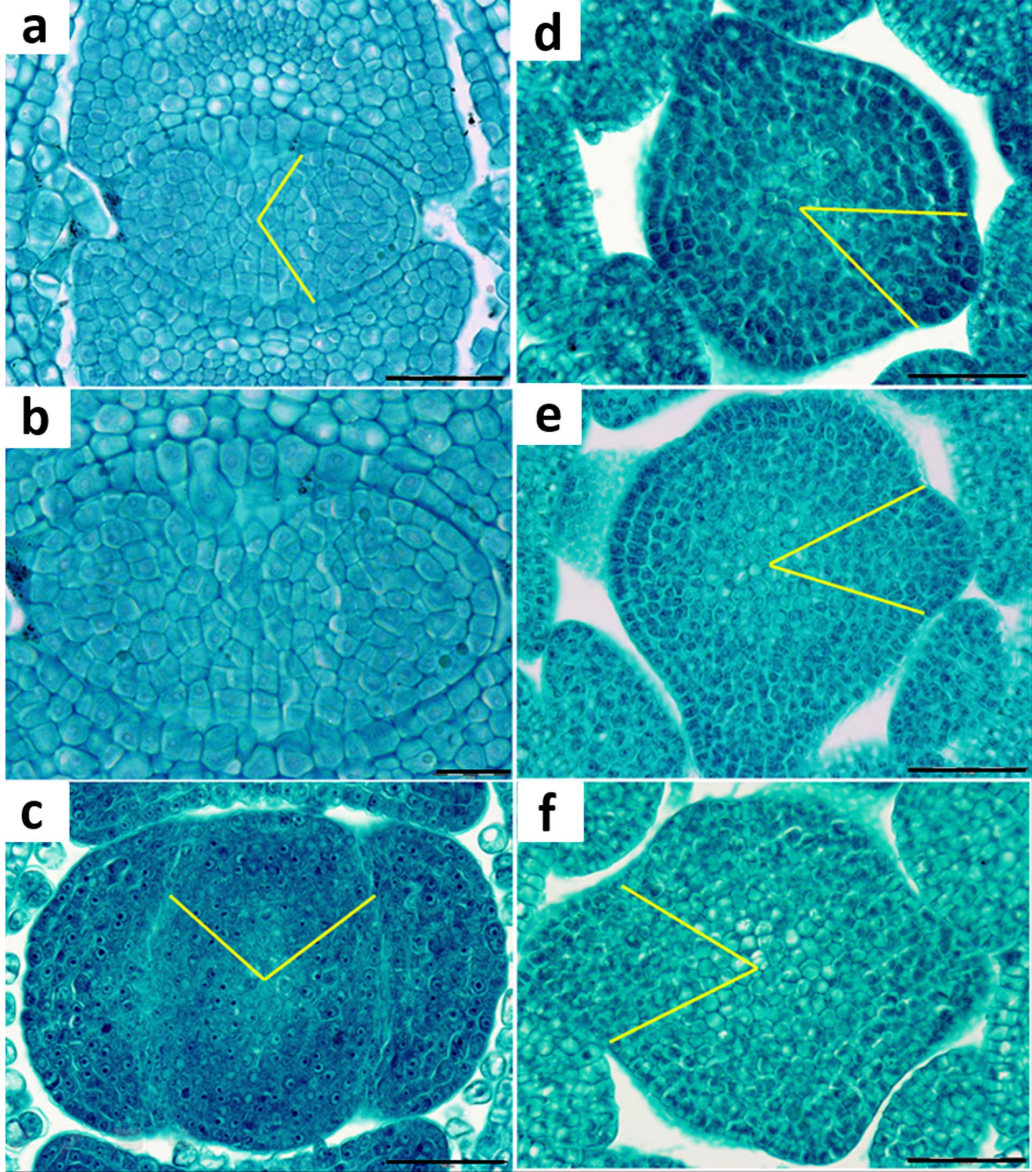
Organization of *Verbena officinalis* shoot apex in vegetative (**a**-**c**) and reproductive (**d**-**f**) phases of development; the size of the lateral organ primordium relative to the size of the apical meristem is shown in the angular measure (yellow lines); the apex in **b** is the same as in **a** but magnified to expose the cellular pattern of the meristem surface already polarized to form the next pair of primordia; scale bar in **a**, **c**-**f** is 50 µm, that in **b** is 20 µm

Secondary transitions, during which primordia identity was maintained, were unpredictable. In some axes, the first helical pattern continued unchanged until the end of a growing season, while in others, it quickly transformed. The course of these transitions varied (Table 2). Altogether, seven various phyllotactic patterns resulting from all types of transitions were identified, and many different sequences of their transformations were recorded (Tables 1, 2, 3, Figs. 2, 3, 4). The prevailing and most stable was the main Fibonacci pattern (Fig. 3c). Less frequent were the main bijugy (Fig. 3b) and Lucas patterns (Fig. 2a). Both of them, once formed, often continued along the axis for many nodes. Also noted was a rare second accessory pattern (Figs. 2b, 3a) resulting from multiple secondary transitions, often transforming further into bijugy through the addition of one parastichy in the set of five (Fig. 3d). Despite the general prevalence of helical patterns, the transient states of tricussate and tetracussate phyllotaxis were frequent in the secondary transitions and seemed to be important for the chirality of the subsequent helical pattern. For instance, the direct transitions between the main Fibonacci and Lucas patterns always proceeded with reversal of the ontogenetic helix. However, in the case of multiple transitions involving a transient tricussate pattern, both chiral configurations of the resulting Lucas phyllotaxis occurred with the same frequency. The frequency of a particular phyllotactic pattern depended very much upon the order of its appearance in the sequence of secondary multiple transitions. Thus, the first patterns in the sequence, main Fibonacci and Lucas, were the most abundant and final second accessory pattern was the least frequent (Table 3). Every year, the plants from the Przecławice population repeated the characteristic trait of secondary phyllotactic transitions (Table 3). In contrast, the plants of Croatian origin, without exception, showed only the primary transitions, resulting in a Fibonacci pattern in all florescences.

The diameters of ten vegetative and eight florescence apices were measured and the relative sizes of their 20 and 18 primordia were calculated, based on the leaf or bract insertion angle, as the percentage of the apex circumference. Reproductive apices were less variable in size (120–150 µm in diameter) than the vegetative ones (76–156 µm). Only primary transitions were associated with an obvious change in relative primordium size (Fig. 4). In the vegetative apices with the decussate pattern, the relative primordia size was 30% on average and similar for the apices of different sizes (Fig. 4a–c). The relative average size of bract primordia was 15% and no clear differences in this parameter were detected among the apices with different phyllotaxis. Bract insertion angles measured on the apices shown in Fig. 4 were 43° for 4:5 s accessory pattern (Fig. 4d), 44° for 3:4 Lucas (Fig. 4e), and 58° for 2:4 bijugy (Fig. 4f).

### Vascular system architecture in phyllotactic patterns and transitions

**Fig. 5:**
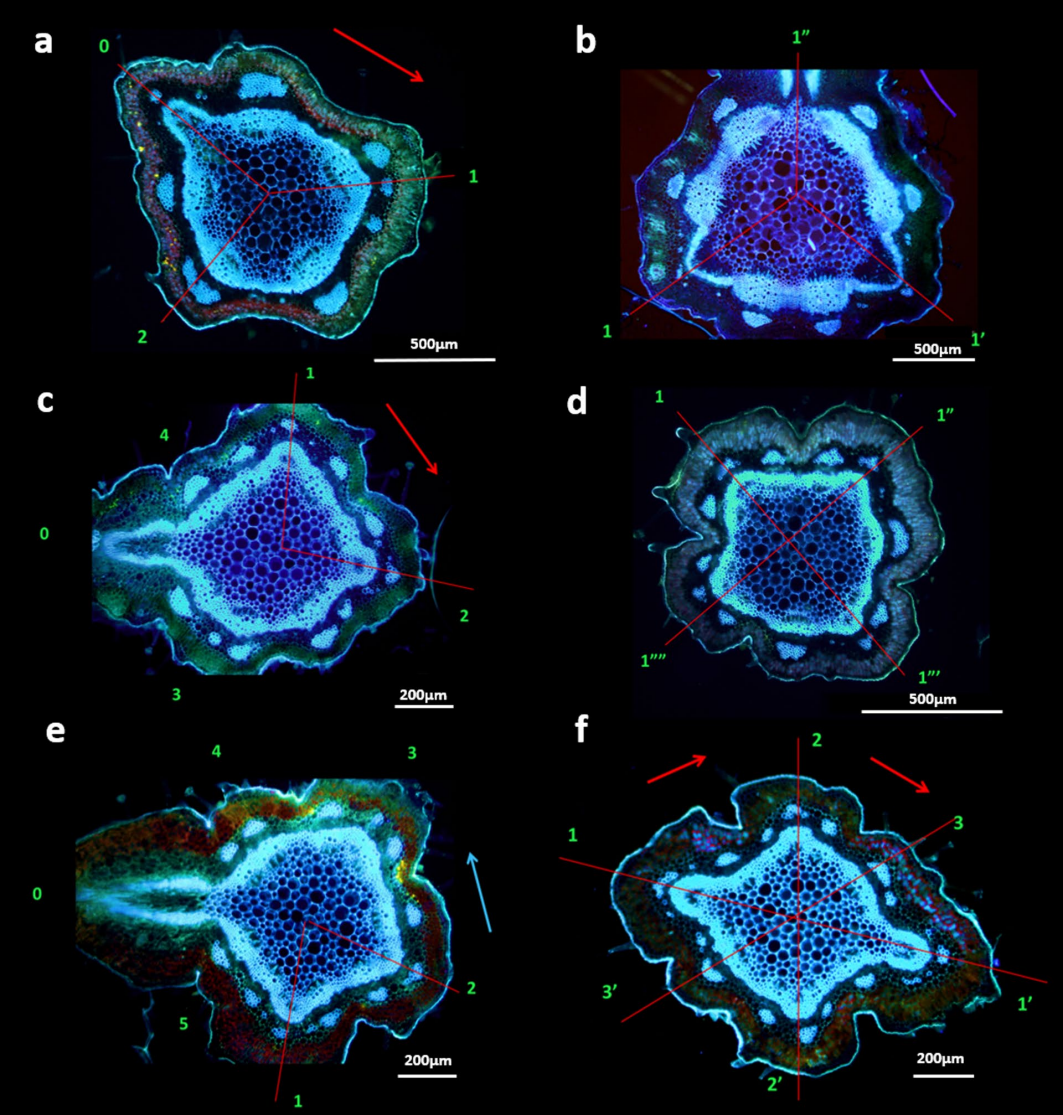
Fluorescent images of well-defined phyllotactic patterns present in six different florescences of common verbena shoots sectioned live transversely and photographed from the bottom side; green numbers mark circumferential positions of consecutive bracts; outer arrows show the direction of the ontogenetic helix in chiral patterns—clockwise in blue and counterclockwise in red; the red thin lines are the rays of the divergence angle between bracts relevant for pattern identification—the consecutive are in a spiral and the neighboring are in a whorl of the whorled pattern. **a**—2z:3 s main Fibonacci phyllotaxis with a ± 137° divergence angle, five vascular sympodia, and three bracts initiated on one revolution of the ontogenetic helix; **b**—3:3 whorled tricussate phyllotaxis with six sympodia and three bracts in one node; **c**—3 s:4z Lucas pattern with the divergence angle ± 99°, seven sympodia and four bracts on one revolution of the ontogenetic helix; **d**—4:4 whorled tetracussate phyllotaxis with eight sympodia; **e**—4z:5 s second accessory pattern with the divergence angle ± 78° and five bracts along the circumference; **f**—2z:4 s main bijugy with ± 69° divergence angle between the consecutive pairs of bracts and ten vascular sympodia

**Fig. 6:**
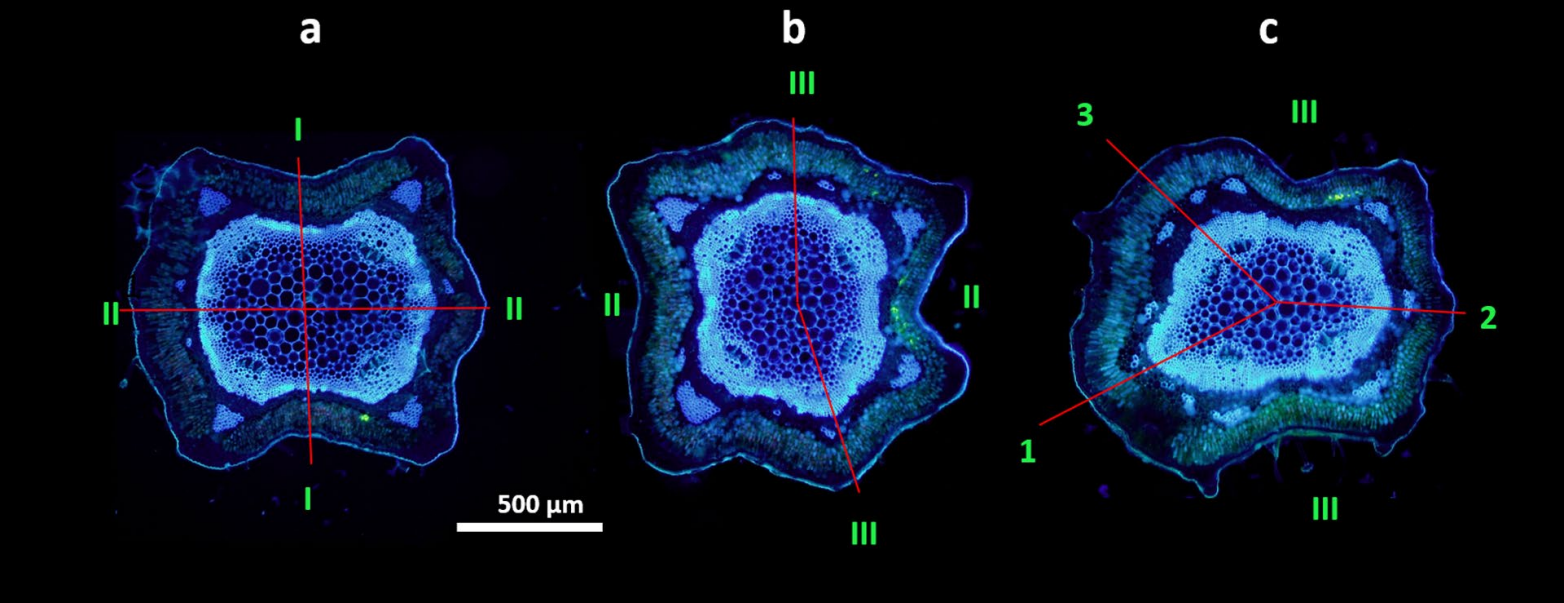
Developmental changes in the vascular system of one verbena shoot during typical primary transition from the vegetative decussate to the main Fibonacci phyllotaxis. The initial symmetric pattern of four vascular bundles representing four vascular sympodia (**a**) changes when the number of bundles increases to five (**c**). The asymmetry of the system is clear before departure of the third (III) and last pair of the lateral organs (**b**). Green Roman letters refer to the elements of the decussate, and the Arabic letters refer to those of the spiral pattern. Red thin lines connect the center of the shoot with the positions of lateral organs on the shoot circumference. Scale bar—500 µm, the same for all three images

**Fig. 7:**
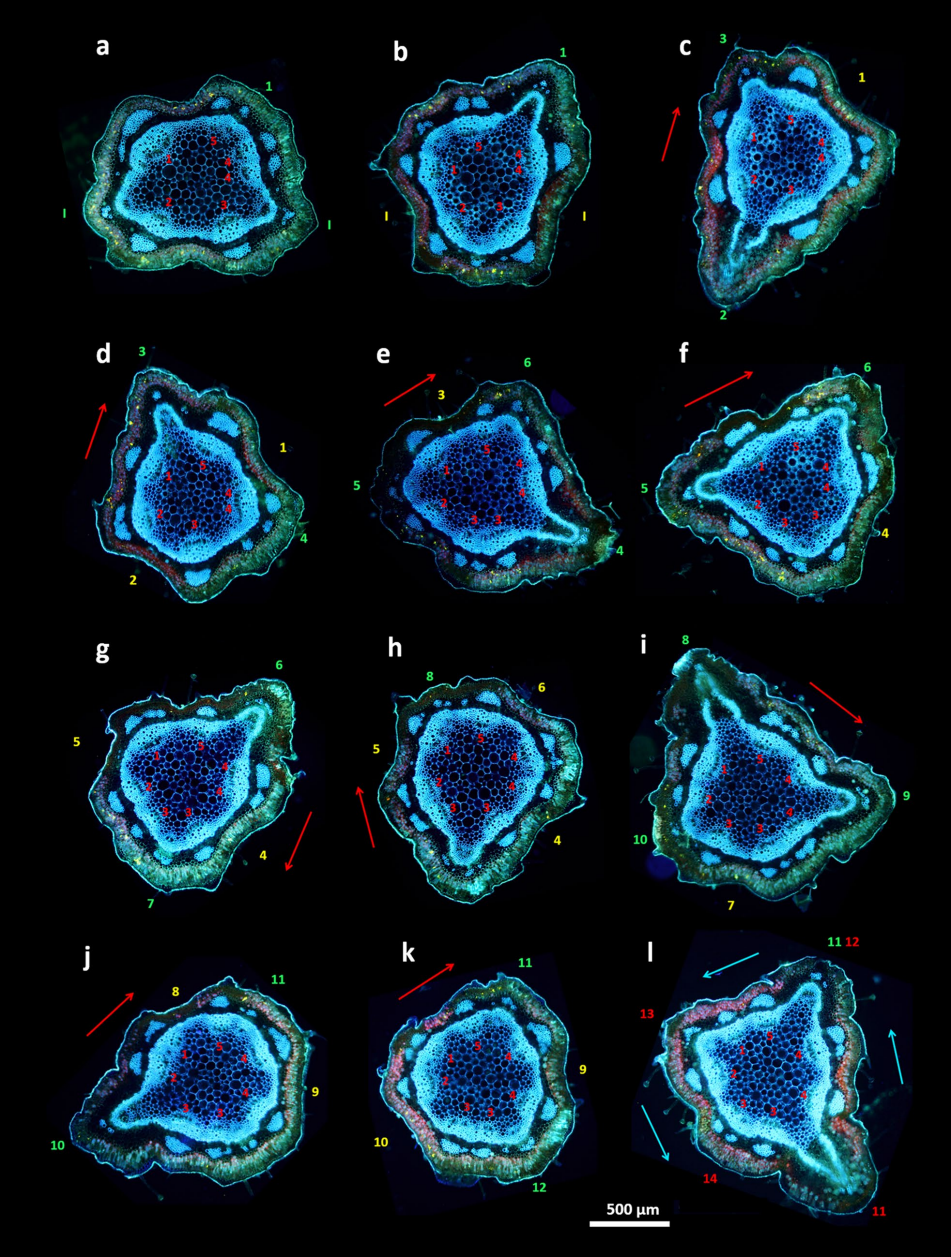
Developmental series of handmade live transverse sections shows reorganization of the vascular system preceding two consecutive phyllotactic transitions in one verbena shoot. Prior to primary transition from the decussate pattern to the main Fibonacci phyllotaxis, one of the initial vascular bundles, representing four vascular sympodia in the vegetative phase of development, is already split into four new bundles (**a**); one of them (number 5) replaces the vascular trace departing to the bract, representing first element of the main Fibonacci pattern (**b**), while the presence of other two (4 and 4) is an initial symptom of the future transition to a Lucas pattern; the transition takes place late, when the 11th vascular trace departs into another position than that anticipated for Fibonacci phyllotaxis (**l**). The increase from five to seven vascular sympodia precedes the secondary phyllotactic transition by splitting the bundle 3 below the 4th node (**e**) and the ultimate separation of the two original bundles, 4 and 4. Despite the presence of seven vascular sympodia, typical for the Lucas pattern, the superficial main Fibonacci pattern is dominant until the 11th node. The outer red arrows point out the consecutive pattern elements (labeled green and yellow) and, thus, the direction of the ontogenetic helix. The helix runs counterclockwise (Z) in the main Fibonacci pattern and changes its course to clockwise in the Lucas pattern, as shown by the outer blue arrows. The orientation of five functional vascular sympodia in the main Fibonacci pattern is in this context opposite to that of the seven in the Lucas pattern (before 1z:2s:3z:5s, after 1s:3z:4s:7z). This can be seen in the positions of departing vascular traces, relative to their sympodial replacements, changing from the right to the left side. Scale bar—500 µm, the same for all images

In light of the above-mentioned result, which suggests the stability of the relative size of bract primordia, the possible dynamics of *Verbena*-shoot vascular structure were examined. The vascular system, composed of leaf traces assembled into sympodia, was closely related to phyllotaxis (Figs. 5, 6, 7). In shoots with decussate phyllotaxis, it was closed, with four vascular bundles representing four vascular sympodia (Fig. 6a). The primary transition to spiral phyllotaxis opened the vascular system through a sectorial change in the number of sympodia from four to five in the main Fibonacci pattern (Figs. 5a, 6c). The increase in the number of vascular bundles could already be seen below the last pair of opposite lateral elements, either foliage leaves or bracts (Figs. 6c, 7a). The addition of one bundle was observed in the shoots with a subsequently stable Fibonacci pattern (Fig. 6). More bundles at this stage predicted secondary transitions (Fig. 7). The number of sympodia increased from five to six in tricussate shoots (Fig. 5b) and to seven in those with Lucas phyllotaxis (Figs. 5c, 7l). The shoots with 4:5 contact parastichies marking the presence of the second accessory pattern showed nine vascular sympodia (Fig. 5e). During the secondary transition leading from this pattern to the main bijugy, the sympodia number increased to ten (Fig. 5f). However, the main bijugy resulting from the primary transition (Table 3) had fewer sympodia (four or six). In the shoots with the main Fibonacci pattern perpetuated for many nodes, a change from five sympodia to eight was noted. These situations indicated developmental increases in the expression of phyllotaxis at the apex.

The continuously sectioned fragment of the shoot, in which phyllotaxis had transformed from the main Fibonacci with five sympodia to Lucas pattern with seven sympodia, showed that the first changes in the vascular system had taken place much earlier than the change in phyllotaxis (Fig. 7). The same sectorial multiplication of vascular bundles below the level of secondary transition was observed in many shoots, regardless of the quality of patterns involved.

### Computer modeling

**Fig. 8:**
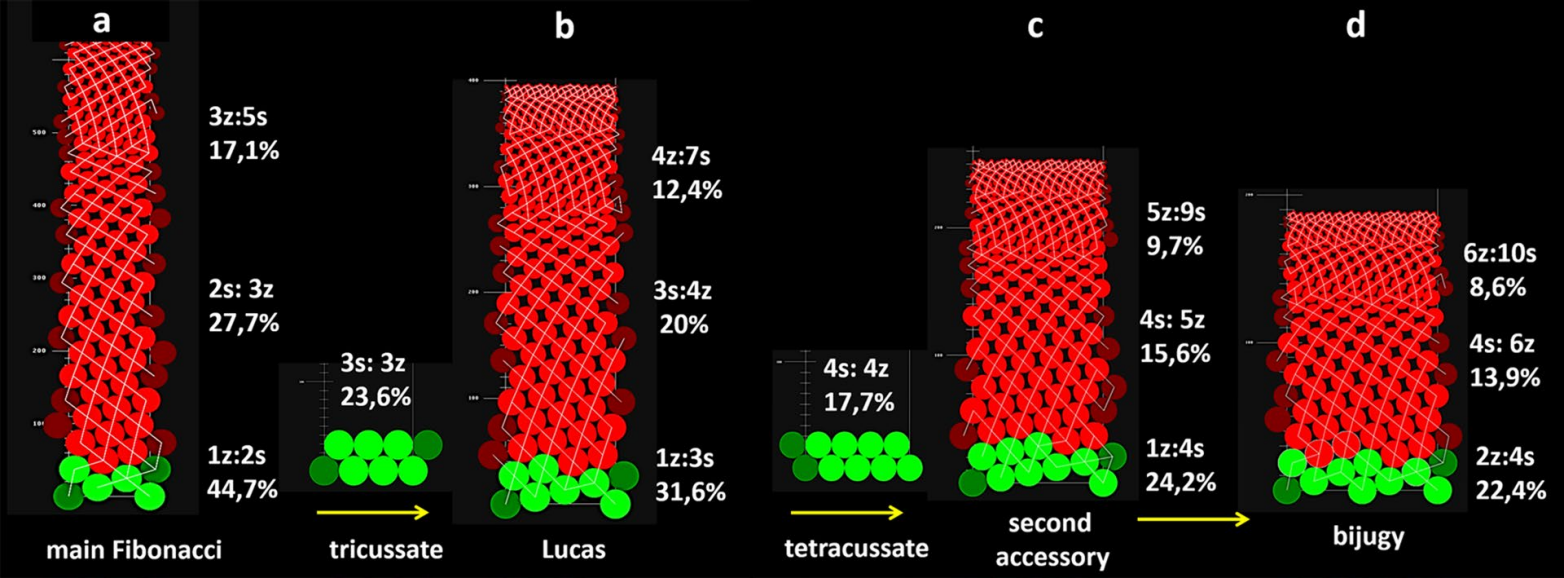
The effects of the continuous decrease in the relative size of primordia (red circles) within each of the common verbena spiral patterns: main Fibonacci (**a**), Lucas (**b**), second accessory (**c**), and main bijugy (**d**). Contact parastichy numbers (as:bz) increase when the primordia radius decreases by 1% in each step within a rectangular simulation space of constant width equal to 100 units and an infinite length measured in units of the same size. Relative primordia sizes are given as percentages. The ranges of their change among the patterns overlap at some developmental stages. The initial spiral patterns and intermediate whorled patterns are shown in green. The cylindrical surface is split open, and thus, the same primordia can be seen, in bright and dull colors, on the opposite sides of the split line. Yellow arrows point out the direction of developmental phyllotactic transitions between the patterns

The sequences of qualitative secondary transitions of phyllotaxis (Table 2) and the direction of developmental changes in the number of vascular sympodia in verbena inflorescence (Figs. 5, 6, 7) suggested a progressive decrease in the size of bract primordia on the apical meristem relative to its circumference. This, however, was not supported by the empirical data gathered on the geometric relationships between primordia and apices in different states of phyllotaxis. To understand these contradictory results, the assumption of a very small change in primordia size was implemented in computer simulations. When the radius of the consecutive primordia decreased by only 1% in each step, the expression of phyllotactic patterns, i.e., the number of contact parastichy pairs, increased (Fig. 8). A comparison of different verbena spiral patterns showed that the ranges of the change in relative primordia size at some developmental stages overlapped. This might facilitate transitions between the patterns and explain why there was not a big variation in the relative bract primordia size measured in real apices (Fig. 4). Running the same simulations for the whorled patterns revealed their transitions to bi-, tri-, and tetrajugate patterns. Subsequently, their expression changed in a similar way to other spiral patterns. Neither trijugy nor tetrajugy was detected in vivo*,* perhaps because of the transient character of the whorled patterns in verbena florescence. The infrequent appearance of bijugy in the primary transitions occurring in vivo (Table 3) supported the observation that between the vegetative and reproductive phases of growth, lateral organ primordia changed in relative size rather dramatically, not slowly and continuously as required for bijugy emergence.

**Fig. 9:**
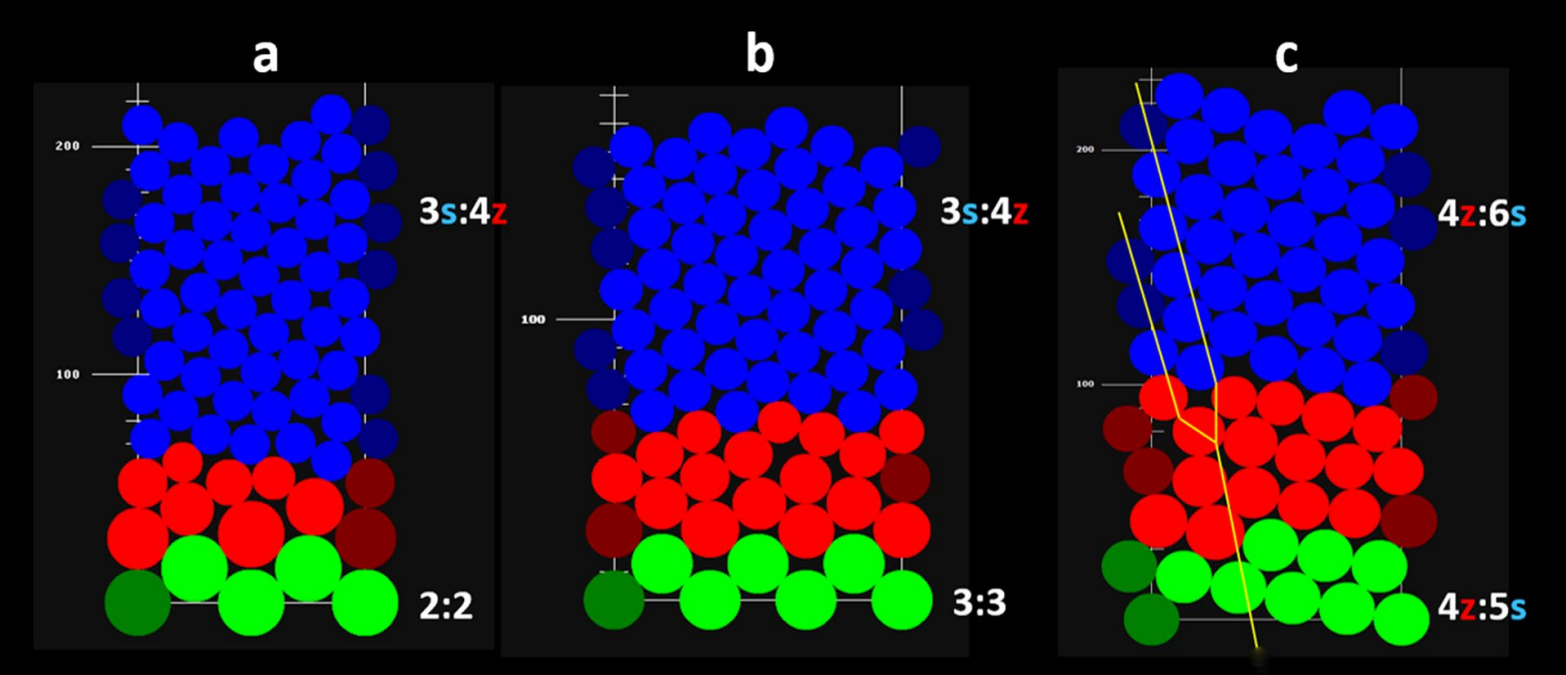
*Verbena officinalis* phyllotactic transitions recreated in computer simulations. **a**, **b**—two primary transitions from the decussate (2:2) and tricussate (3:3) phyllotaxis into the Lucas pattern (3:4); **c**—secondary transition from the second accessory pattern (4:5) to main bijugy (4:6), the same as in Fig. 3d. The initial pattern is shown in green color, the transition zone in red, and the final pattern in blue. Parastichy bifurcation is marked with thin yellow line (**c**). The circular organ primordia radius decreases in size at different rates in the red areas (8 primordia by 7% for each step in **a**, 16 by 3% in **b** and 16 by 1% in **c**), and in green and blue areas, their size is constant. They are packed into a rectangular simulation space, the width of which represents size of the organogenic surface of the apical meristem. The cylindrical surface is split open, and thus, the same primordia can be seen, in bright and dull colors, on the opposite sides of the split line

By performing numerous in silico experiments and applying various changes in primordia size, all primary transitions and most of the secondary transitions were received (Figs. 9, S1). In one variant of the virtual primary transition, the initial decussate pattern required eight primordia to continuously decrease in radius, by 7% in each step, to transform into Lucas phyllotaxis (Fig. 9a). A slower rate of this change, by only 3%, was required to obtain the same result for the initial tricussate pattern (Fig. 9b). These experiments were only the first step toward more precise systemic modeling of verbena phyllotaxis in the future, which would require more quantitative data from the plant material. The in silico experiments showed, however, what kind of data should be sought. For instance, the direct in vivo transitions between the Fibonacci and Lucas patterns always occurred with the reversal of the ontogenetic helix. Under the assumption of a continuous decrease in primordia size, it was impossible to transform the main Fibonacci pattern at all. The pattern in such situation only increased in expression (Fig. 8). To overcome this and other limitations, some special parameters of the simulations, such as vertical tolerance in positioning the primordia in the first available space or subtle fluctuations in their changes in size, needed to be added. The secondary transition between the second accessory pattern and bijugy (Fig. 3d) was, for instance, obtained with the application of these special parameters (Fig. 9c).

### Verbena culture

**Fig. 10:**
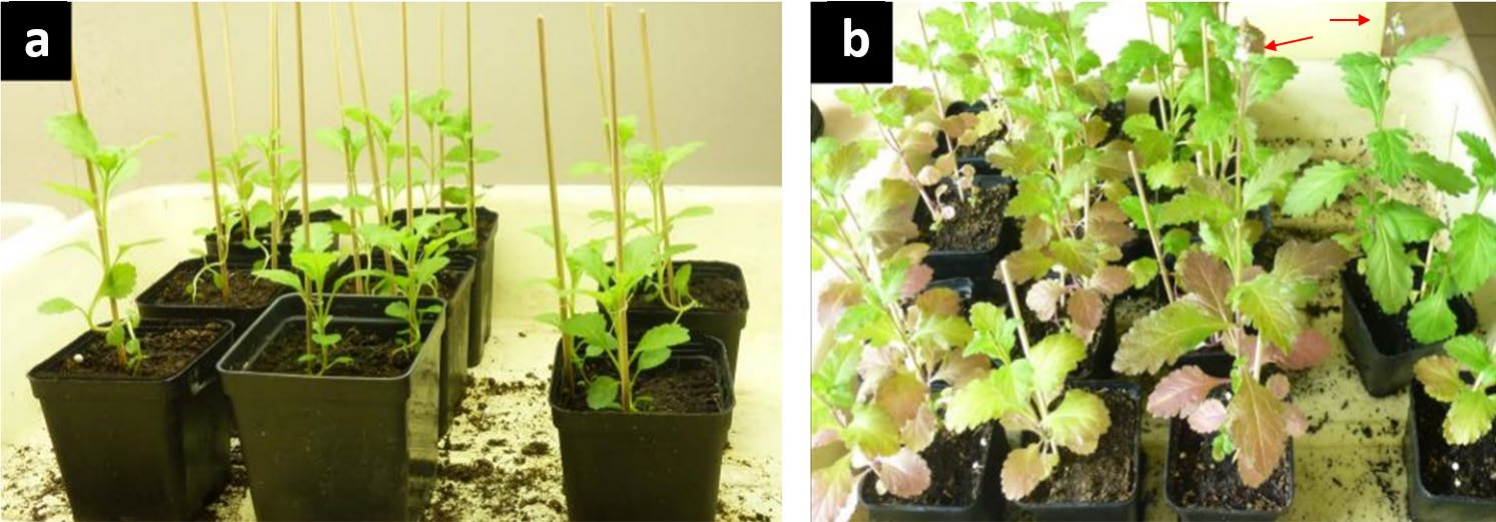
*Verbena officinalis* grown in the controlled environment of the growth chamber. **a**—in the short day the plants remain in the vegetative state; **b**—the long day induces flowering. Red arrows show emerging terminal main florescences

Long-term observations showed that the plants from the Przecławice population propagated from seeds repeated the same pattern of phyllotactic diversity, indicating genetic control of the trait. Differences between individual plants (Table 3) and between Polish and Croatian populations supported this presumption. It was necessary to develop a protocol for growing plants in the controlled environment, which is essential for the proposed model plant. The preliminary experiments showed that the seeds sown in soil with pH 5.5–6.5 and kept in a growth chamber with a short day (8 h) at 21 °C and 120-μmol m^−2^ s^−1^ light intensity, germinated in seven days. In those subjected to a long day (16 h), germination did not occur. The plants, after germination, remained in the vegetative stage for 6 weeks in the short day (Fig. 10a) and then had to be transferred to the long day conditions, which induced a new, reproductive phase of growth. They ultimately flowered in 5–6 weeks (Fig. 10b), closing their life cycle in three to four months. In this setup, all experimental treatments modifying plant growth and development became possible. However, phyllotaxis of plants from the culture is yet to be characterized and compared with that of the plants from the field. The ability to grow in a controlled environment, short life cycle, and production of hundreds small seeds by each individual plant make *Verbena officinalis* similar to the other model plant, *A. thaliana*. The unique phyllotactic diversity and vegetative propagation of this plant are its additional and especially attractive traits.

## Discussion

The alteration of phyllotaxis during plant ontogeny is now of particular interest owing to the discovery of the genetic mechanisms regulating stem cell proliferation, SAM size, and primordia size—factors responsible for the qualities of phyllotactic patterns (Landrein et al. 2015; Willis et al. 2016). However, it has long been known that some mutations affect the number of initiated organ primordia, altering the geometric parameters of the apex. The *perianthia* (*pan*) mutant of *A. thaliana*, instead of having four canonic sepals, four petals, and six stamens, exhibits five floral organs in each of the three initial whorls of the flower. This effect is most likely due to the diminishing size of the perianth primordia in the first two whorls and the increasing size of the stamen primordia, because the size of the meristem does not change in *pan* mutants (Running and Meyerowitz 1996). The double mutation of *CYCLOIDEA* and *DICHOTOMA*, the two genes expressed on the adaxial (dorsal) side of the floral meristem of *Antirrhinum majus*, leads to the development of a peloric flower with sixfold symmetry of the corolla, whereas the WT plants are pentameric (Luo et al. 1996). The lack of *CLAVATA 1* and *CLAVATA 3* genes control over *WUSCHEL* gene activity intensifies cell proliferation and increases the apical meristem size (Clark et al. 1993, 1995, 1997; Durbak and Tax 2011; Laux et al. 1996). Larger meristems than those in WT plants also develop in the rice loss‐of‐function mutants of the *DECUSSATE* (*DEC*) gene (Itoh et al. 2012) due to enhanced cell divisions. This leads to the development of decussate phyllotaxis instead of the typical distichous. The same effect of altered phyllotaxis was earlier observed and defined as the ABERRANT PHYLLOTAXIS (ABPHYL) syndrome in maize (Greyson and Walden 1972; Greyson et al. 1978). Decussate shoot meristems of maize were subsequently also shown to be larger in *abphyl1* mutants than in WT plants (Jackson and Hake 1999).

The sequences of multiple phyllotactic transitions in *Verbena officinalis* shoots suggest a constant change in the ratio between primordia size and the size of the organogenic surface of the apical meristem, and this has been confirmed in the case of primary transitions. The most intriguing, however, is that in the secondary transitions, unusual patterns emerge from the main Fibonacci pattern, which developmentally should be the most stable (Szpak and Zagórska-Marek 2011). The reason why it is so dynamic in *Verbena* remains a mystery. The secondary transitions do not result from such obvious reconstruction of geometric relations at the apex that takes place between the vegetative and reproductive phase of growth. The primordia of numerous bracts are of the same identity, and in *Verbena*, significant differences in their size could not be detected among phyllotactic patterns, possibly due to the fact that the available sample of apices was small. It is also puzzling that the phyllotactic pattern in verbena florescence is sometimes stable and sometimes changing, and that the same initial pattern may give rise to three different patterns. A potential reason could be the cumulative effect of irregularities in the initiation process. This possibility is supported by our earlier results of computer simulations (Zagórska-Marek and Szpak 2016) and those presented in this work on the virtual secondary transition between the second accessory pattern and bijugy (Fig. 9c). It involved very small changes in primordia size, but required the application of tolerance parameters. Another possibility yet to be investigated is the role of the vascular system in sending putative acropetal signals that might modify phyllotaxis.

The *Verbena officinalis* model appears to be especially useful for future, more detailed studies on the relationship between organogenesis, which takes place at the shoot apical meristem, and differentiation of the primary vascular system. The present results, based on the analysis of already elongated axes, show that the changes in the vascular system greatly precede the appearance of the dedicated lateral organ. New vascular sympodia may be observed well before a change in phyllotaxis, which is concordant with the classical observations made by Larson in cottonwood (Larson 1975, 1977); this should direct more attention to the role of the acropetal differentiation of procambial strands (Dengler 2006; Esau 1965). This developmental process may be more important than previously thought. The verbena case (Fig. 7) demonstrates not only the multiplication of vascular sympodia before phyllotactic transition but also the changing sides of the leaf trace connections related to the new sympodia orientation in future Lucas phyllotaxis. Moreover, the direction of multiple secondary transitions seems to be controlled by the process of adding new vascular sympodia one by one in a sequence: 4—5—6—7—8—9—10. This promotes the adequate sequence of phyllotactic patterns: decussate—Fibonacci—tricussate—Lucas—tetracussate—second accessory—bijugy.

The present results may contribute to deciphering the role of the vascular system in regulating meristem organogenic activity. The significance of that role has been revealed by the close examination of mutation effects of *PIN-FORMED1* (*PIN1*) gene in *A. thaliana*. The slow differentiation of vascular strands in *pin 1* mutant keeps pace with apex growth and organogenic activity at the rosette stage, when internodes are short. This allows formation of the foliage leaves to occur as well as it does in the WT plant. Bolting of the inflorescence in the next phase of growth, however, makes the vascular strands lag behind the departing apex, which, in their absence, is not capable of producing lateral organ primordia (Banasiak 2010). Another example is *Torreya*; the delayed differentiation of vascular strands below its apex allows for setting up a new chiral configuration of the phyllotactic pattern every year. The pattern, in principle, does not change from year to year in other conifers, which have their vascular strands close to the apex, actively laying down primordia, as in *Abies* or *Picea* (Banasiak and Zagórska-Marek 2006). The problem of pre-procambium differentiation is discussed by Dengler (2006). She admits that acropetal signaling from the future vascular tissues is not resolved because the expression pattern of the important marker gene *ARABIDOPSIS THALIANA HOMEOBOX GENE 8 (ATBH-8*), is yet to be understood. New possibilities open up with the discovery of some proteins, such as the DNA-binding with one finger transcription factors (Dof TFs) family, being engaged in a very early signaling network, allowing the cells of the ground tissue to acquire pre-procambial identity (Le Hir and Bellini 2013). It is much too early, however, to employ these methods in the verbena model.

Until now, common verbena has been analyzed in the botanical literature mostly from a taxonomic point of view (Martínez et al. 1996; O’Leary and Múlgura 2014) and as a medicinal plant (Cao et.al 2012; Deepak and Handa 2000a, b; Lai et al. 2006; Liu et al. 2012; Makino et al. 2009,). The present report reveals the additional virtues of the species. The potential value of this plant has been shown here for further basic studies on the mechanisms of plant growth and development. Molecular-genetic methods may even be employed in the future. Screening for “phyllotactic mutants” in *A. thaliana* so far has been unsuccessful, and this plant’s phyllotaxis is quite uniform. This is why another model plant is needed. *Verbena* phyllotaxis is dynamic, and this trait seems to have a genetic foundation, as shown by the comparison of Silesian populations with those from Croatia. There are also clear differences in the level of phyllotactic diversity among individual plants. Some other advantages of *Verbena officinalis*, which is proposed to become a model system, make it similar to *A. thaliana,* even for the molecular-genetic studies. These traits are as follows: short life cycle, indeterminate growth of florescence, innumerous small and viable seeds (hundreds per one inflorescence in one growing season), and easy seed germination. Finally, the plant can be grown in controlled conditions (Chmiel 2015), and in contrast to *A. thaliana*, once-induced mutants may be propagated vegetatively and maintained in culture. Interestingly, *Verbena* has already been a subject of molecular studies. It appears to have been involved in two independent intergeneric chloroplast transfers during the evolutionary radiation of *Verbena* complex in American continents (Yuan and Olmsted 2008).

In Christine Beveridge’s words, “it is a pity there are so many interesting phenomena in plant development outside *Arabidopsis thaliana*” (personal communication). *Magnolia*, *Torreya*, and *Abies balsamea* are some examples of plants with dynamic phyllotaxis that are unsuitable for experimental work. *Verbena officinalis* may one day allow the elucidation of the molecular mechanisms that underly the developmental instability of phyllotaxis.

## Supplementary Information

Below is the link to the electronic supplementary material.

Fig. S1. Three panels providing detailed information on the parameters used in computer simulations to receive phyllotactic transitions shown in Fig.9 (DOC 244 KB)

## References

[CR1] Banasiak A (2010) Putative dual pathway of auxin transport in organogenesis of *Arabidopsis*. Planta 233:49–61. 10.2307/43564742. https://www.jstor.org/stable/43564742 10.1007/s00425-010-1280-0 20886230

[CR2] Banasiak A, Zagórska-Marek B (2006). Signals flowing from mature tissues to shoot apical meristem affect phyllotaxis in coniferous shoot. Acta Soc Bot Pol.

[CR3] Battey NH, Lyndon RF (1990) Reversion of flowering. Bot Rev 56:162–189. 10.2307/4354144. https://www.jstor.org/stable/4354144

[CR4] Cao G, Cong XD, Zhang Y, Cai BC, Liu Z, Xu Z, Zhou H (2012). Simultaneous determination of four bioactive compounds in *Verbena officinalis* L. by using high-performance liquid chromatography. Pharmacogn Mag.

[CR5] Chmiel K (2015) Architecture dynamics of the vascular system in inflorescent shoots of *Verbena officinalis*. Dissertation. University of Wroclaw

[CR6] Clark SE, Running MP, Meyerowit EM (1993). CLAVATA1, a regulator of meristem and flower development in *Arabidopsis*. Development.

[CR7] Clark SE, Running MP, Meyerowitz EM (1995). CLAVATA3 is a specific regulator of shoot and floral meristem development affecting the same processes as CLAVATA1. Development.

[CR8] Clark SE, Williams RW, Meyerowitz EM (1997). The *CLAVATA1* gene encodes a putative receptor kinase that controls shoot and floral meristem size in *Arabidopsis*. Cell.

[CR9] Deepak M, Handa SS (2000). Antiinflammatory activity and chemical composition of extracts of *Verbena officinalis*. Phytother Res.

[CR10] Deepak M, Handa SS (2000). Quantitative determination of the major constituents of *Verbena officinalis* using high performance thin layer chromatography and high pressure liquid chromatography. Phytochem Anal.

[CR11] Dengler NG (2006). The shoot apical meristem and development of vascular architecture. Can J Bot.

[CR12] Douady S, Couder Y (1996). Phyllotaxis as a dynamical self-organizing process Part II: the spontaneous formation of a periodicity and the coexistence of spiral and whorled patterns. J Theor Biol.

[CR13] Durbak AR, Tax FE (2011). CLAVATA signaling pathway receptors of *Arabidopsis* regulate cell proliferation in fruit organ formation as well as in meristems. Genetics.

[CR14] Esau K (1965). Vascular differentiation in plants.

[CR15] Gola E (1996). Phyllotaxis diversity in *Lycopodium clavatum* L. and *Lycopodium annotinum* L. Acta Soc Bot Pol.

[CR16] Gola EM, Banasiak A (2016). Diversity of phyllotaxis in land plants in reference to the shoot apical meristem structure. Acta Soc Bot Pol.

[CR17] Greyson RI, Walden DB (1972). The ABPHYL syndrome in *Zea mays*. I. Arrangement, number and size of leaves. Amer J Bot.

[CR18] Greyson RI, Walden DB, Hume AJ (1978). The ABPHYL syndrome in *Zea mays*. II. Pattern of leaf initiation and the shape of the shoot apical meristem. Can J Bot.

[CR19] Itoh J-i, Hibara K-i, Kojima M, Sakakibara H, Nagato Y (2012). Rice *DECUSSATE* controls phyllotaxy by affecting the cytokinin signaling pathway. Plant J.

[CR20] Jackson D, Hake S (1999). Control of phyllotaxy in maize by the *ABPHYL1* gene. Development.

[CR21] Kang J, Tang J, Donelly P, Dengler NG (2003). Primary vascular pattern and expression of *ATHB-8* in shoots of *Arabidopsis*. New Phytol.

[CR22] Lai SW, Yu MS, Yuen WH, Chang RC (2006). Novel neuroprotective effects of the aqueous extracts from *Verbena officinalis* Linn. Neuropharmacology.

[CR23] Landrein B, Refahi Y, Besnard F, Hervieux N, Mirabet V, Boudaoud A, Vernoux T, Hamant O (2015). Meristem size contributes to the robustness of phyllotaxis in *Arabidopsis*. J Exp Bot.

[CR24] Larson PR (1975). Development and organization of the primary vascular system in *Populus deltoides* according to phyllotaxy. Am J Bot.

[CR25] Larson PR (1977). Phyllotactic transitions in the vascular system of *Populus deltoides* Bartr. As determined by C^14^ labeling. Planta.

[CR26] Laux T, Mayer KFX, Berger J, Jurgens G (1996). The *WUSCHEL* gene is required for shoot and floral meristem integrity in *Arabidopsis*. Development.

[CR27] Le Hir R, Bellini C (2013). The plant-specific dof transcription factors family: new players involved in vascular system development and functioning in *Arabidopsis*. Front Plant Sci.

[CR28] Liu Z, Xu Z, Zhou H, Cao G, Cong XD, Zhang Y, Cai BC (2012). Simultaneous determination of four bioactive compounds in *Verbena officinalis* L. by using high-performance liquid chromatography. Pharmacogn Mag.

[CR29] Luo D, Carpenter R, Vincent C (1996). Origin of floral asymmetry in *Antirrhinum*. Nature.

[CR30] Makino Y, Kondo S, Nishimura Y, Tsukamoto Y, Huang ZL, Urade Y (2009). Hastatoside and verbenalin are sleep-promoting components in *Verbena officinalis*. Sleep Biol Rhythms.

[CR31] Martínez S, Botta S, Múlgura ME (1996). Morfología de las inflorescencias en Verbenaceae-Verbenoideae I: Tribu Verbeneae. Darwiniana.

[CR32] Müller-Xing R, Clarenz O, Pokorny L, Goodrich J, Schubert D (2014). Polycomb-group proteins and *FLOWERING LOCUS T* maintain commitment to flowering in *Arabidopsis thaliana*. Plant Cell.

[CR33] O'Leary N, Múlgura M (2014). Synopsis of tribe Verbeneae Dumortier (Verbenaceae) in Peru. Phytotaxa..

[CR34] Peaucelle A, Couder Y (2016). Fibonacci spirals in a brown alga [*Sargassum muticum* (Yendo) Fensholt] and in a land plant [*Arabidopsis thaliana* (L.) Heynh.]: a case of morphogenetic convergence. Acta Soc Bot.

[CR35] Running MP, Meyerowitz EM (1996). Mutations in the PERIANTHIA gene of *Arabidopsis* specifically alter floral organ number and initiation pattern. Development.

[CR36] Staedler YM, Weston PH, Endress PK (2007). Floral phyllotaxis and floral architecture in *Calycanthaceae* (Laurales). Int J Plant Sci.

[CR37] Szpak M, Zagórska-Marek B (2011). Phyllotaxis instability—exploring the depths of first available space. Acta Soc Bot Pol.

[CR38] U.S. National Plant Germplasm System [Internet] (2020). https://npgsweb.ars-grin.gov/gringlobal/taxonomydetail.aspx?id=41164. Accessed 31 Aug 2020

[CR39] Weberling F (1983). Fundamental features of modern inflorescence morphology. Bothalia.

[CR40] Willis L, Refahi Y, Wightman R, Landrein B, Teles J, Casey Huang K, Meyerowitz EM, Jönsson H (2016). Cell size and growth regulation in *Arabidopsis*. Proc Natl Acad Sci.

[CR41] Wróblewska M, Dołzbłasz A, Zagórska-Marek B (2016). The role of ABC genes in shaping perianth phenotype in the basal angiosperm *Magnolia*. Plant Biol J.

[CR42] Yin X, Meicenheimer R (2016). The ontogeny, phyllotactic diversity, and discontinuous transitions of *Diphasiastrum digitatum* (Lycopodiaceae). Am J Bot.

[CR43] Yin X, Lacroix C, Barabé D (2011). Phyllotactic transitions in seedlings: the case of *Thuja occidentalis*. Botany.

[CR44] Yuan YW, Olmstead RG (2008). A species-level phylogenetic study of the Verbena complex (Verbenaceae) indicates two independent intergeneric chloroplast transfers". Mol Phylogenet Evol.

[CR45] Zagórska-Marek B (1985). Phyllotactic patterns and transitions in *Abies balsamea*. Can J Bot.

[CR46] Zagórska-Marek B (1994). Phyllotaxic diversity in *Magnolia* flowers. Acta Soc Bot Pol.

[CR47] Zagórska-Marek B, Szpak M (2008). Virtual phyllotaxis and real plant model cases. Funct Plant Biol.

[CR48] Zagórska-Marek B, Szpak M (2016). The significance of γ-and λ-dislocations in transient states of phyllotaxis: how to get more from less—sometimes!. Acta Soc Bot Pol.

[CR49] Zagórska-Marek B, Sokołowska K, Turzańska M (2018). Chiral events in developing gametophores of *Physcomitrella patens* and other moss species are driven by an unknown, universal direction-sensing mechanism. Am J Bot.

