# Supplementary material for: Verbena officinalis Verbenaceae (Lamiales): a new plant model system for phyllotaxis research
Source: J Plant Res. 2021 Apr 8;134(3):441–56. doi: 10.1007/s10265-021-01288-2 (PMC8106610; doi:10.1007/s10265-021-01288-2)
Supplement: Supplementary file 1 — Fig. S1. Three panels providing detailed information on the parameters used in computer simulations to receive phyllotactic transitions shown in Fig.9 (DOC 244 KB) [file 10265_2021_1288_MOESM1_ESM.doc]

**Electronic supplementary materials**

**Title:**

***Verbena officinalis* Verbenaceae (Lamiales) - a new plant model system for phyllotaxis research**.

**Authors:**

Beata Zagórska-Marek PhD

Magdalena Turzańska MSc

Klaudia Chmiel MSc

**Journal:**

Journal of Plant Research

**Corresponding author:**

Beata Zagórska-Marek PhD, University of Wrocław, Kanonia Str 6/8, 50-328 Wrocław, Poland

Tel: +48-71-3754094

Fax: +48-71-3754118

E-mail: beata.zagorska-marek@uwr.edu.pl

**Content:**

**Fig. S1**

**
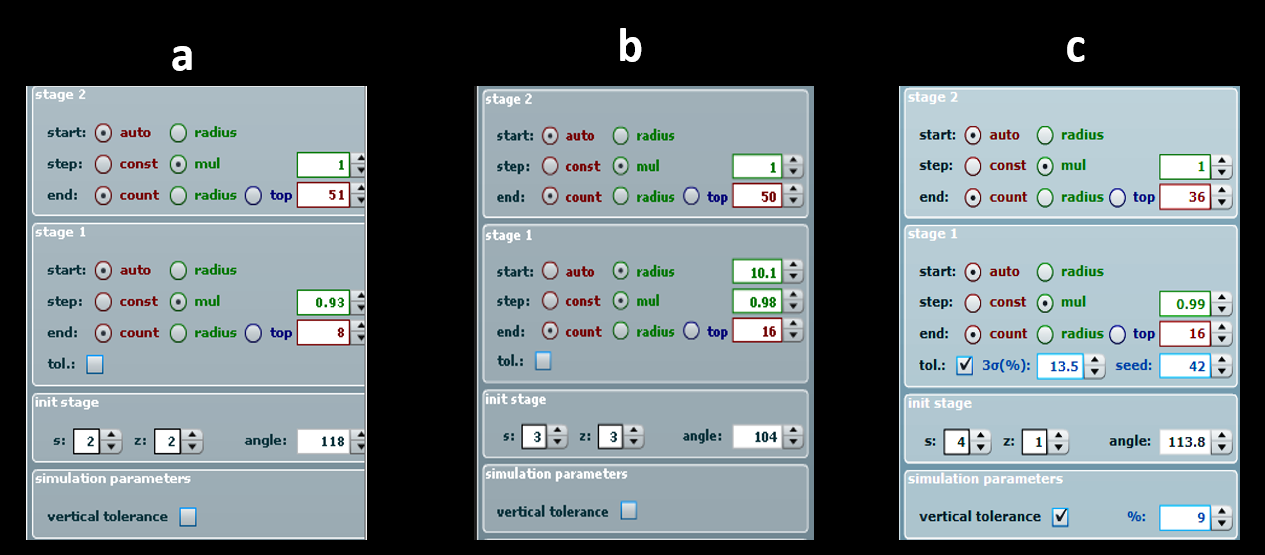
**

Three panels providing detailed information on the parameters used in computer simulations to receive phyllotactic transitions shown in Fig.9
